# Supplementary material for: DMRforPairs: identifying Differentially Methylated Regions between unique samples using array based methylation profiles
Source: BMC Bioinformatics. 2014 May 15;15:141. doi: 10.1186/1471-2105-15-141 (PMC4046028; doi:10.1186/1471-2105-15-141)
Supplement: Additional file 2 — DMRforPairs output for the comparison of A431-MCF7 and NA17018-NA17105. Please start from the HTML files in each folder. Available via the BMC Bioinformatics website. [file 1471-2105-15-141-S2.zip › 1394847754114233_MOESM2_ESM/A431_MCF7/figures/17873.pdf]

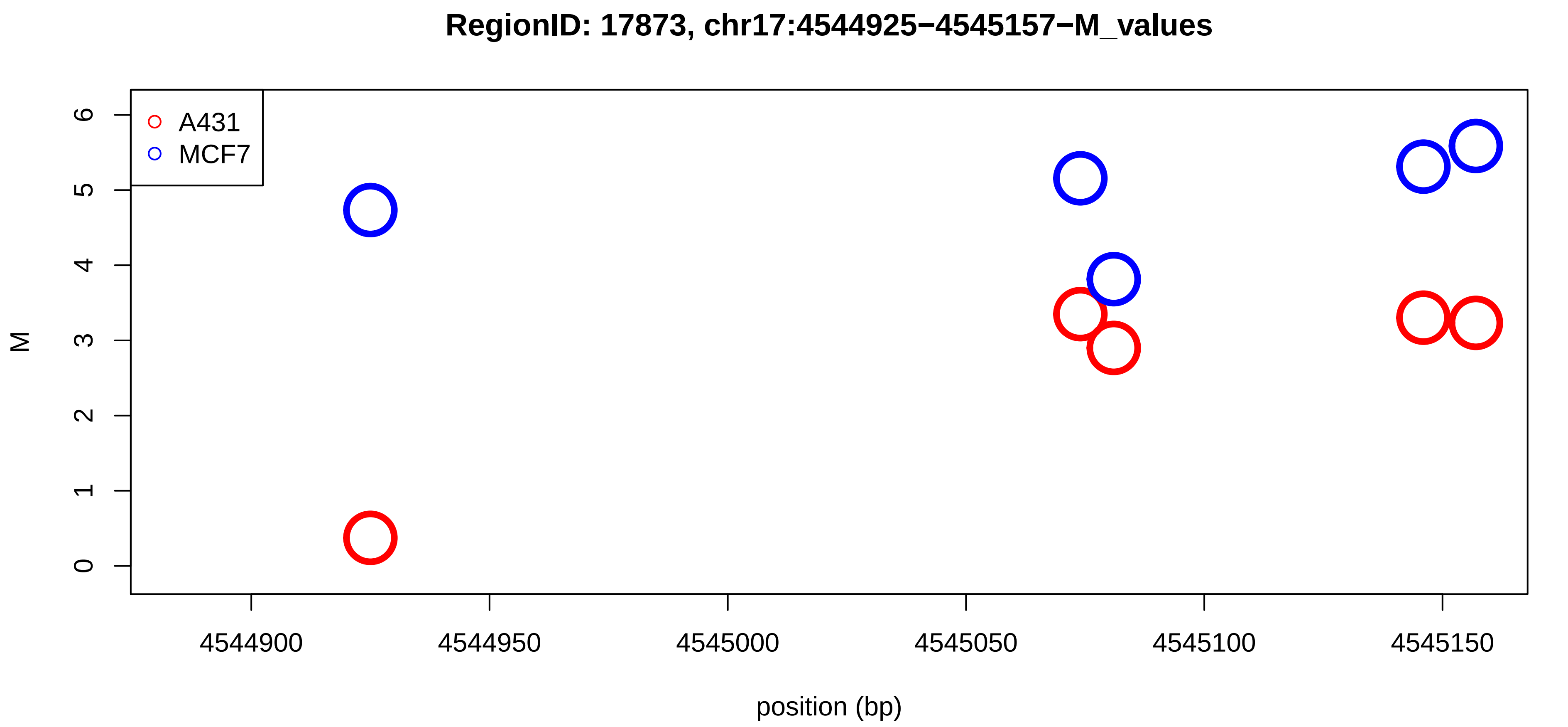

RegionID: 17873, chr17:4544925−4545157−M_values
A431
MCF7
M
0
1
2
3
4
5
6
4544900
4544950
4545000
4545050
4545100
4545150
position (bp)

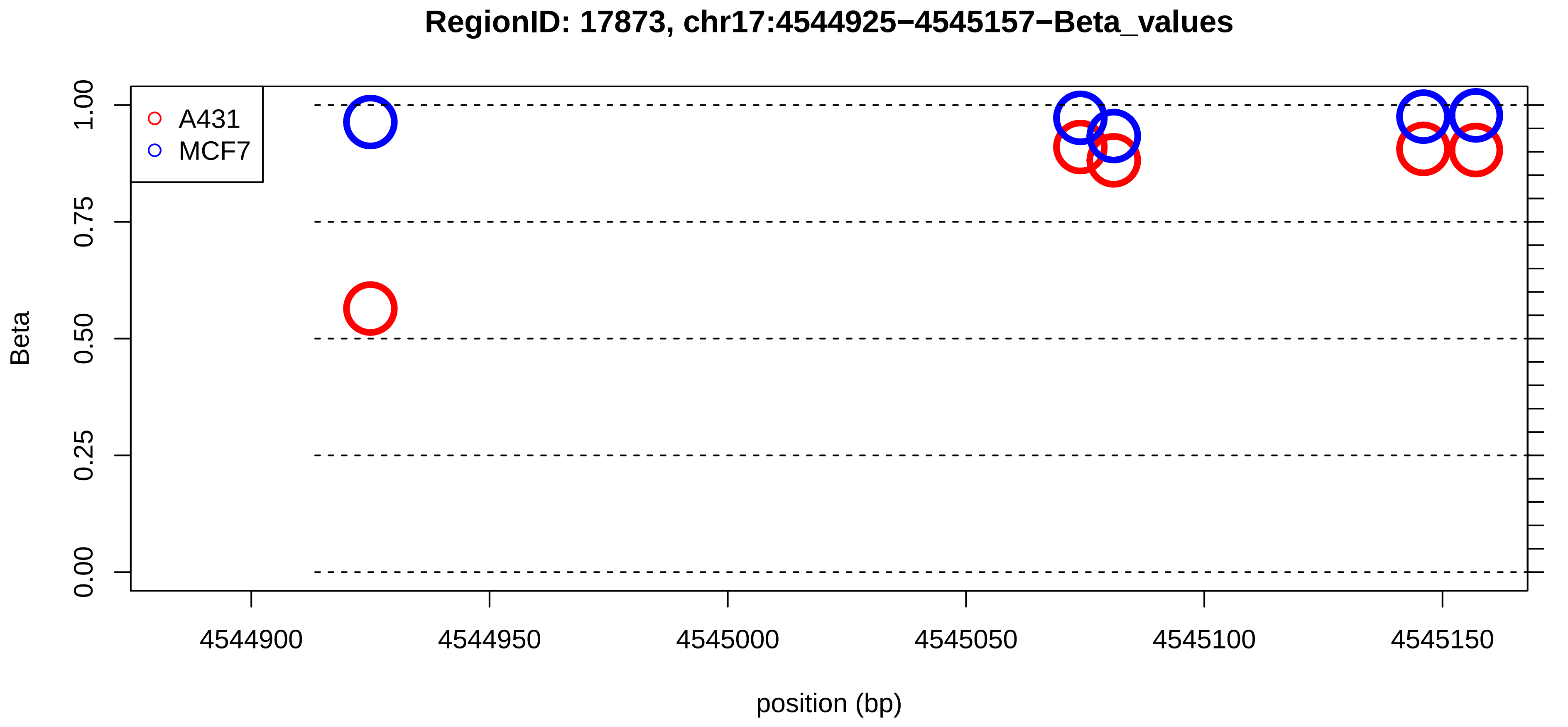

RegionID: 17873, chr17:4544925−4545157−Beta_values
A431
MCF7
Beta
0.00
0.25
0.50
0.75
1.00
4544900
4544950
4545000
4545050
4545100
4545150
position (bp)

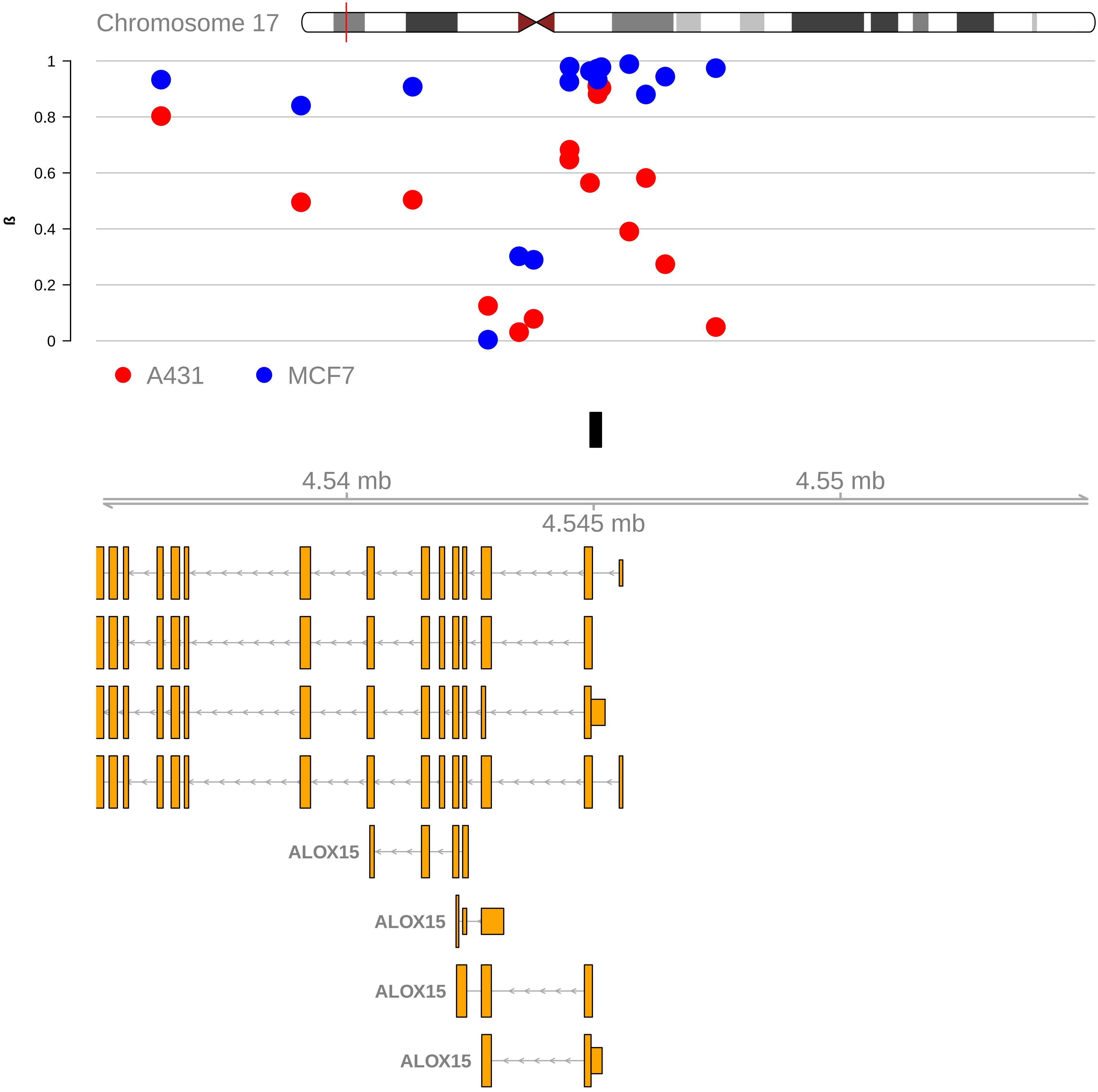

Chromosome 17
1
0.8
0.6
0.4
0.2
0
ß
A431
MCF7
4.54 mb
4.545 mb
4.55 mb
ALOX15
ALOX15
ALOX15
ALOX15
